# The Consumption of Spirits

**Published:** 1887-10-01

**Authors:** R. R. Redmayne

**Affiliations:** House Governor to the Royal Infirmary, Newcastle-On-Tyne


### STATISTICS OF ALCOHOL.

### THE CONSUMPTION OF SPIRITS OF WINE.

By R. R. Redmayne,
House Governor to the Royal Infirmary, Newcastle-on-Tyne.

In your last number I notice your table of comparative costs for stimulants, alcohol, etc., in various hospitals at different dates, and I see you introduce "spirits of wine" as an element in the comparison. I think this point should be made somewhat more clear, if your table is intended for information on the consumption of stimulants as such. I know that in our own case the introduction of the antiseptic method has had a great influence on the consumption of spirit, as we use it as the fuel for our spray producers.

In an average year we use ten times as much methylated spirit for fuel as we do rectified spirit for the dispensary. We charge the latter under "drugs," as it goes into tinctures, which we mostly make ourselves. The former goes under antiseptic and other dressings. You will see that a source of considerable error may therefore be found in such a return as you propose, partly from want of showing the purpose to which the spirit is applied, and partly from the different plans dispensaries may have of providing their medicines. At times we also use a large quantity of spirit for steam-kettles, etc. You will observe therefore that a very large proportion of our expense in the matter of alcohol is quite apart from the question of the use of alcohol in hospitals as popularly understood.

